# Assessment of Clinical Utility of Assaying FGF-23, Klotho Protein, Osteocalcin, NTX, and Sclerostin in Patients with Primary Hyperparathyroidism

**DOI:** 10.3390/jcm10143089

**Published:** 2021-07-13

**Authors:** Monika Sykała, Piotr Szumowski, Małgorzata Mojsak, Saeid Abdelrazek, Łukasz Żukowski, Danuta Lipińska, Ilona Juchnicka, Gabryela Kozłowska, Małgorzata Szelachowska, Adam Krętowski, Janusz Myśliwiec

**Affiliations:** 1 Department of Nuclear Medicine, Medical University of Białystok, M. Skłodowskiej-Curie St. 24A, 15-276 Białystok, Poland; piotrmjs@wp.pl (P.S.); mni@o2.pl (M.M.); saeid@op.pl (S.A.); lukzuk85@gmail.com (Ł.Ż.); janusz.mysliwiec69@gmail.com (J.M.); 2 Department of Endocrinology, Diabetology and Internal Medicine, Medical University of Białystok, M. Skłodowskiej-Curie St. 24A, 15-276 Bialystok, Poland; lipinska.danuta11@gmail.com (D.L.); ilona.sikora06@gmail.com (I.J.); gabryelakozlowska@gmail.com (G.K.); mszelachowska@poczta.onet.pl (M.S.); adamkretowski@wp.pl (A.K.)

**Keywords:** primary hyperparathyroidism, parathyroid, parathyroidectomy, FGF-23, Klotho, osteocalcin, NTX, sclerostin

## Abstract

The purpose of this study was to assess the clinical usefulness of assaying the fibroblast growth factor (FGF-23), Klotho, osteocalcin, N-terminal telopeptide of type I collagen (NTX), and sclerostin levels in patients with primary hyperparathyroidism (PHPT) as markers of bone damage as well as for surgical treatment success. Seventeen patients with hypercalcemic PHPT and normal kidney function were studied. In all patients, PTH (parathormone), serum calcium, and creatinine were performed before and six months after parathyroidectomy (PTX). The studied group included patients whose PTH and calcium concentrations normalized post-operatively and with confirmed histopathological diagnosis. The control group consisted of nine age-matched healthy volunteers. The PHPT patients had elevated concentrations of FGF-23, osteocalcin, and NTX and reduced levels of sclerostin, as compared to the control group. After PTX, osteocalcin, NTX, and sclerostin levels normalized. The plasma values of FGF-23 decreased significantly, but remained higher than in healthy subjects. Serum Klotho protein levels did not differ significantly in the two groups. These results suggest that osteocalcin and NTX may potentially be considered as markers of PHPT progression. Additionally, serum normalization of osteocalcin, NTX, and sclerostin might be considered as indicators of PTX success. On the other hand, FGF-23 can represent a parameter reflecting the degree of calcium–phosphate imbalance in PHPT patients, but its usefulness in monitoring the effects of PTX requires further research. The clinical utility of assaying Klotho in PHPT remains to be confirmed.

## 1. Introduction

### Primary Hyperparathyroidism

Primary hyperparathyroidism (PHPT) is one of the main reasons for hypercalcaemia. It is usually caused by a solitary adenoma of the parathyroid gland and is characterised by an elevated or inappropriately normal level of parathormone (PTH) [1,2]. The decision to treat PHPT surgically is not only made on the basis of biochemical indicators, but also depends on the severity of clinical symptoms. Parathyroidectomy (PTX) is indicated for all symptomatic PHPT patients. It is regarded as the only definitive therapy that mitigates the risk of nephrolithiasis and improves bone mineral density. Nowadays, only about 20–30% of PHPT patients present classical symptoms of hyperparathyroidism [3,4]. Many patients have non-specific or mild symptoms, which are difficult to objectivise and their association with PHPT can only be ascertained after successful surgery [5].

The fibroblast growth factor 23 (FGF-23) is a peptide secreted by the osteocytes and osteoblasts, at present considered to be a regulator of the calcium–phosphate balance and an important element of the FGF-23-bone–kidney axis. FGF-23 enhances urinary phosphate excretion and inhibits the production of the active form of vitamin D. The importance of FGF-23 in PHPT patients has been recently described [6].

Klotho is a protein, which shows the greatest expression in the renal tubules. The αKlotho protein plays the role of a co-receptor for FGF-23. It strengthens its binding with the FGFR (fibroblast growth factor receptor) by forming the FGFR/αKlotho/FGF-23 complex. The soluble form of αKlotho inhibits the activity of 1-α-hydroxylase in the kidneys and affects the secretion of PTH. Expression of Klotho has also been demonstrated in the osteocytes, which may imply its role in osteogenesis [7,8,9,10,11].

PHPT leads to increased bone remodelling in approx. 50–80% cases and may be accompanied by decreased bone mass density, which is associated with a heightened risk of fractures [12]. Measurements of bone turnover factors may be a prognostic element and may correlate with changes in bone mass after effective PTX [13]. Osteocalcin is a marker of bone tissue formation. Synthesised mainly in the osteoblasts, it has a close affinity to hydroxyapatite. N-terminal telopeptide of type I collagen (NTX) is regarded as a marker of bone resorption [14].

Sclerostin is a glycoprotein produced almost exclusively by the osteocytes. It is currently considered to be one of the major factors, which regulate bone remodelling processes. Intense mechanical stimuli result in cessation of sclerostin production by the osteocytes and lead to an increase in the bone mass, while immobilisation boosts its expression in the bones and causes bone mass loss [15].

The purpose of the study was to assess the clinical utility of assaying plasma/serum concentrations of FGF-23, Klotho, osteocalcin, NTX, and sclerostin as potential markers of PHPT progression and the efficacy of PTX.

## 2. Materials and Methods

### 2.1. Study Population

The present study was a retrospective analysis of patients referred to the Department of Nuclear Medicine at the Medical University of Białystok, for parathyroid scintigraphy, in the years 2018–2019. Criteria of selection for the study group included hypercalcaemia (>2.75 mmol/L), elevated serum PTH (>68.3 pg/mL), and eGFR > 70 mL/min/1.73 m^2^ (estimated glomerular filtration rate), as well as confirmed PHPT in histopathological material and normalised concentrations of PTH and calcium after surgery. Exclusion criteria comprised: history of chronic kidney disease, hepatic function disorders, thyrotoxicosis, neoplastic disease, family history of hyperparathyroidism, heart failure, lung disease, use of glucocorticosteroids, biphosphonates, calcimimetics, estrogens, denosumab, lithium carbonate, and thiazide diuretics.

Seventeen patients met the above criteria. Concentrations of PTH, calcium, phosphates, and creatinine in the blood serum of all the patients were measured; USG (ultrasonography) of the neck and scintigraphy of the parathyroids (subtraction scan and SPECT/CT, single-photon emission computed tomography) was performed. In the study group, measurements of serum/plasma FGF-23, Klotho, osteocalcin, NTX, and sclerostin were performed twice: before the surgery and six months after effective PTX. The patients who had been taking vitamin D formulas had been advised to discontinue them for four weeks before their blood samples were taken so as to minimise their impact on the laboratory results. The decision to administer surgical treatment was taken by an endocrinologist on the basis of clinical indications and biochemical parameters (unrelated to the present project). The control group consisted of nine healthy volunteers, matching the study group in terms of gender and age, each of whom had a venous blood sample taken. The project was approved by the Bioethical Committee of the Medical University of Białystok (decision no. R-I-002/175/2019 of 28 February 2019).

### 2.2. Biochemical Examinations

The biochemical examinations were performed using an Abbott Alinity analyzer (Abbott Laboratories, Abbott Park, IL, USA).

PTH concentrations were measured by means of the chemiluminescence method (reference range: 15–68.3 pg/mL), total calcium levels—by the colorimetric method (reference range: 2.25–2.75 mmol/L), phosphates—by the phosphomolibdenum method (reference range: 0.74–1.52 mmol/L), and creatinine—by the enzymatic method (reference range: 0.55–1.02 mg/dL). The eGFR (estimated glomerular filtration rate) was calculated with the simplified MDRD (Modification of Diet in Renal Disease) formula

### 2.3. Imaging Studies

USG examinations were carried out by different operators and using different scanners (the patients provided the results). The scintigraphy images were obtained using the planar technique and SPECT-CT projections with Siemens Symbia Intevo (Siemens Healthineers, Erlangen, Germany). All the scintigraphy images were evaluated by the same physician.

### 2.4. Immunoenzymatic Assays (ELISA)

Blood samples were drawn in the morning (after fasting) into two test tubes: one containing EDTA and the other—a clotting activator. After centrifuging the tubes, plasma and serum were separated into smaller samples and frozen at −80 °C. Once blood had been collected from all patients (before and after surgery), all the samples were defrosted and assays were performed according to the guidelines provided by producers, using the following kits:

- FGF-23: enzyme-linked immunosorbert assay kit for FGF-23 (Cloud-Clone Corp. (Katy, TX, USA); catalogue no. SEA746Hu); minimum detectable concentration <6.1 pg/mL. Coefficient of variability (CV) <10%.
- Klotho protein: human soluble α-Klotho assay kit—IBL (Immuno-Biological Laboratories Co. (Minneapolis, MN, USA); code no. 27998); minimum detectable concentration: 6.15 pg/mL; CV = 2.7%.
- Osteocalcin: enzyme-linked immunosorbert assay kit for Osteocalcin (Cloud-Clone Corp.; catalogue no. SEA471Hu); minimum detectable concentration < 0.264 ng/mL; CV < 10%.
- NTX: Osteomark NTx serum assay (catalogue no. 9021); minimum detectable concentration: 3.2 nM; CV = 4.6%.
- Sclerostin: TECO sclerostin HS kit (TECOmedical Group (Sissach, Switzerland), catalogue no. TE1023 HS), minimum detectable concentration: 0.009 ng/mL; CV = 6%.

### 2.5. Statistical Analysis

Statistical analysis was done with the help of Statistica 13.3 software (TIBCO Software Inc., Palo Alto, CA, USA). Depending on normality of data distribution, parametric or non-parametric tests were applied. Normality of distribution was assessed using the Shapiro–Wilk test. The parametric test used in our analysis was the *t*-Student test; the non-parametric one was the *U* Mann–Whitney test. The optimum cut-off points for the selected parameters were determined by means of ROC (receiver operating characteristic) curves. The relationships between the individual factors were investigated using Spearman’s rank order correlations. The level of statistical significance was set at *p* < 0.05.

## 3. Results

The study group consisted of 17 patients (14 women and three men) with PHPT, aged 28–74 years (mean age: 53.8 years ± 11.9). Their mean body mass index (BMI) was 24.7 kg/m^2^ ± 2.3. The control group comprised nine healthy volunteers (seven women and two men) aged 25–68 years (mean age: 49.7 years ± 12.4; mean BMI: 22.9 kg/m^2^ ± 1.6). The biochemical data for the two groups are presented in Table 1. Seven of the patients with PHPT were diagnosed with nephrolithiasis, four with features of osteopenia or osteoporosis, six with hypertension. Four persons complained of low mood or depressive symptoms, four of muscle or joint pain, two patients suffered from chronic gastritis and duodenitis, and one from anaemia. Two patients had been treated with radioiodine for thyroid disorders (but euthyroid at the time of recruitment). In most cases, the USG results were inconclusive. Scintigraphy showed the presence of parathyroid adenoma in 14 patients. In two persons, the results were inconclusive, while in one, the result was negative.

### 3.1. FGF-23

**Figure 1:**
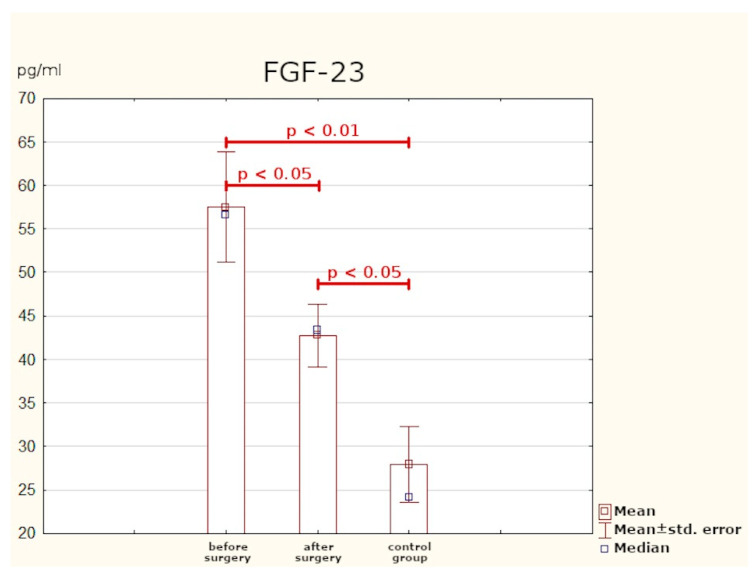
Plasma concentrations of FGF-23.

**Figure 2:**
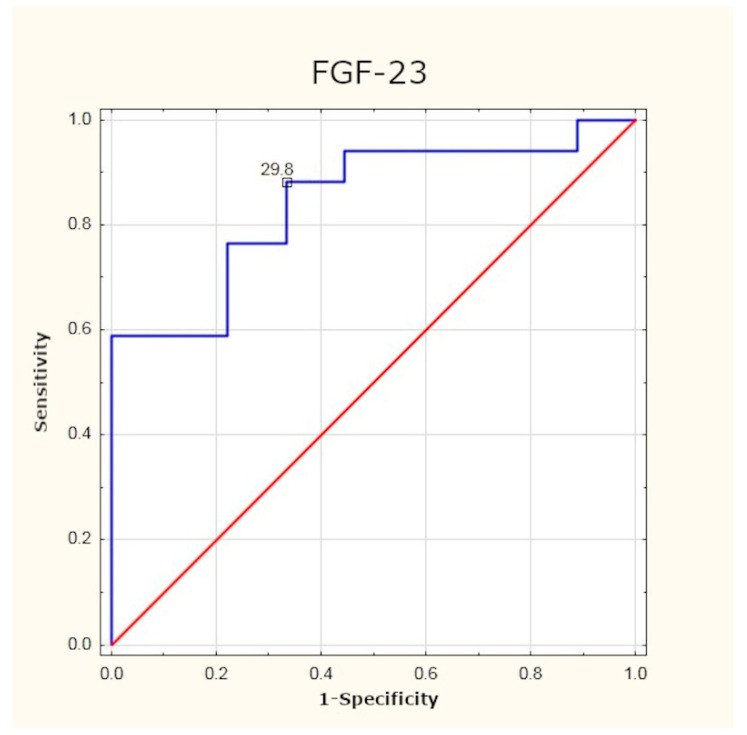
Determination of optimum cut-off point for FGF-23 using ROC (receiver operating characteristic) curve.

In the study group, we observed statistically significantly higher mean concentrations of FGF-3 as compared to the control group. The optimum cut-off point for patients with PHPT was determined to be 29.8 pg/mL (with 88% sensitivity and 67% specificity; *p* < 0.001). After effective surgery, the values of FGF-23 decreased significantly (*p* < 0.05), but did not reach normal levels—the mean concentration of FGF-23 six months post-surgery differed from the mean level found in healthy subjects (Table 2, Figure 1 and Figure 2).

### 3.2. Klotho

**Figure 3:**
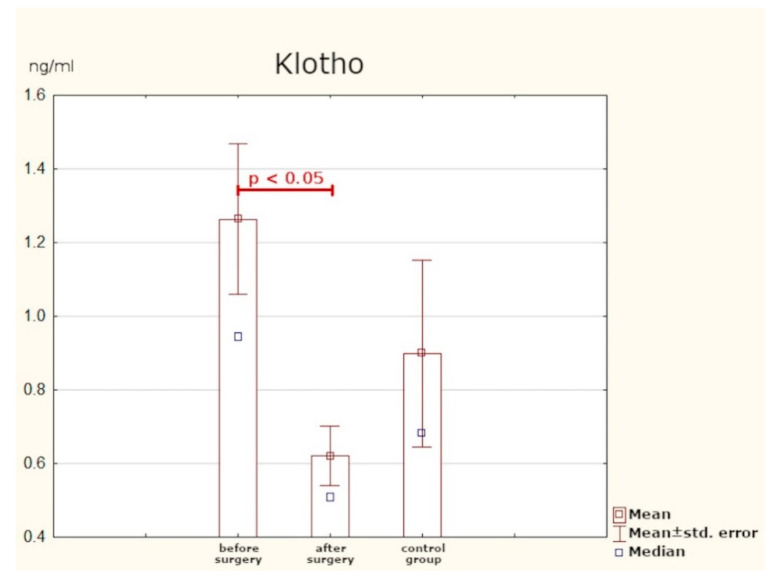
Serum concentrations of Klotho protein.

The preoperative mean concentration of the Klotho protein in the study group was slightly higher than that in the control group, but the difference is not statistically significant. After PTX, a statistically significant decrease in the plasma values of Klotho was observed (Table 3, Figure 3).

### 3.3. Osteocalcin

**Figure 4:**
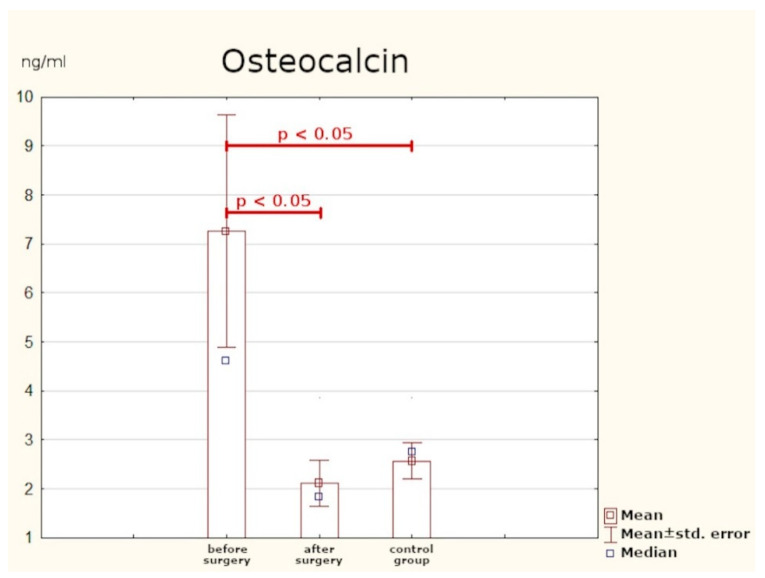
Serum concentrations of osteocalcin.

**Figure 5:**
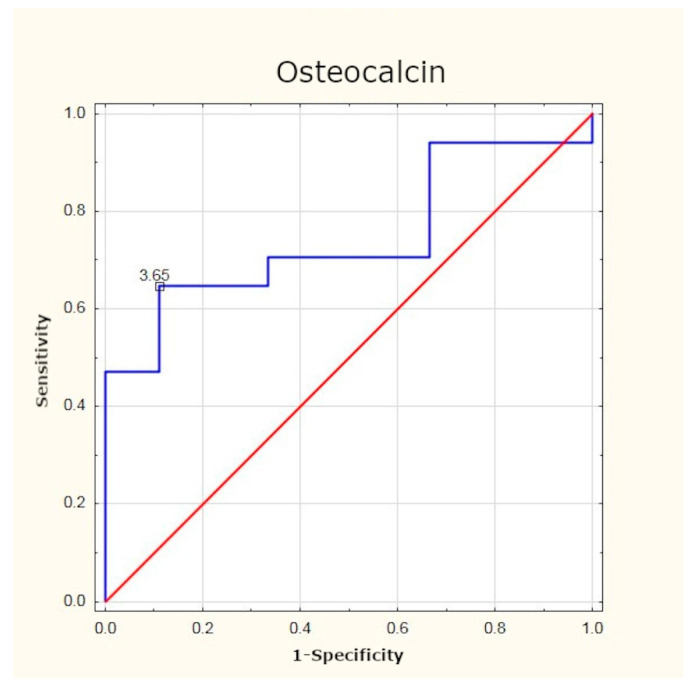
Determination of optimum cut-off point for osteocalcin using ROC curve.

Prior to the surgery, the mean concentration of osteocalcin in patients with PHPT was significantly higher than in the control group. The optimum cut-off point was assessed to be 3.65 ng/mL (sensitivity: 64%; specificity: 89%; *p* < 0.05). Six months after PTX, the values of osteocalcin returned to normal. The mean post-surgery concentration did not significantly differ from the levels found in healthy persons (Table 4, Figure 4 and Figure 5).

### 3.4. NTX

**Figure 6:**
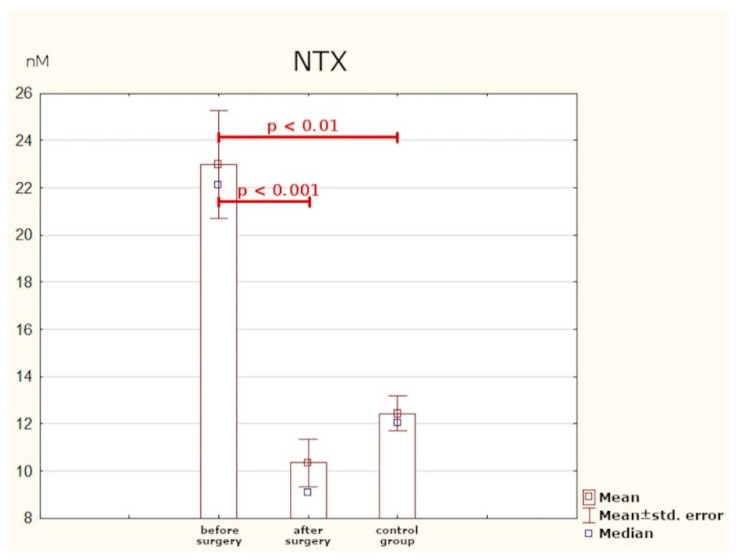
Serum concentrations of NTX.

**Figure 7:**
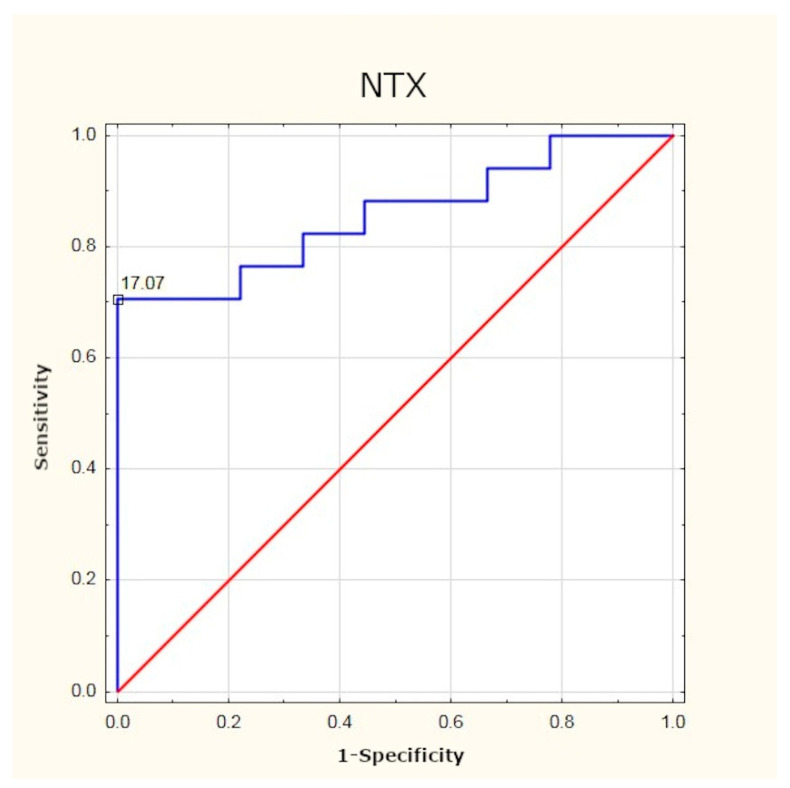
Determination of optimum cut-off point for NTX using ROC curve.

As in the case of osteocalcin, the mean preoperative serum concentration of NTX in the study group was statistically significantly much higher than in the healthy subjects. Here, the optimum cut-off point in PHPT patients was identified as 17.07 nM (sensitivity: 70%; specificity: 100%; *p* < 0.001). After effective surgery, the concentration of the peptide returned to normal (Table 5, Figure 6 and Figure 7).

### 3.5. Sclerostin

**Figure 8:**
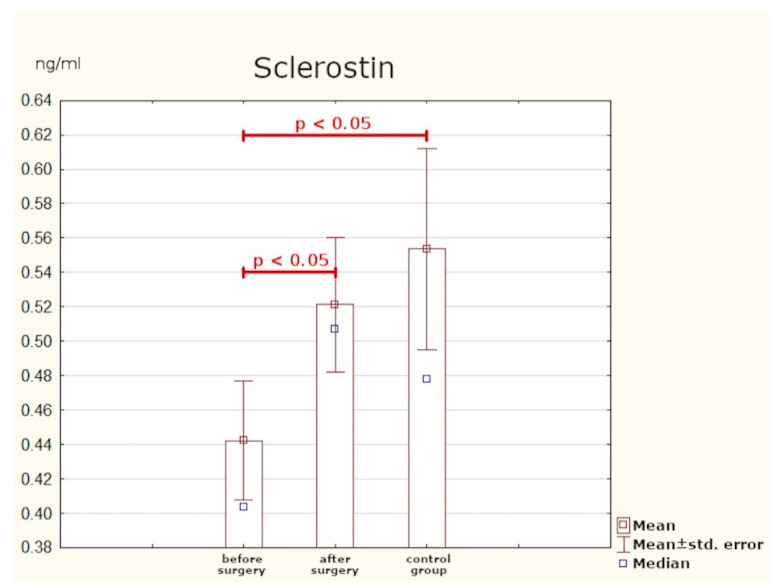
Serum concentrations of sclerostin.

Patients with PHPT were found to have statistically significantly lower serum concentrations of sclerostin as compared with the control group. After surgery, normalisation of this parameter was observed (Table 6, Figure 8).

The analysis of Spearman’s rank correlation coefficients in PHPT patients was performed. There was a moderate, positive correlation between the serum concentration of PTH and calcium (r_S_ = 0.53), a moderate, positive correlation between the values of calcium and osteocalcin (r_S_ = 0.49), and a high, positive correlation between the serum concentration of calcium and NTX (r_S_ = 0.62). The other relationships were not statistically significant (Table 7).

## 4. Discussion

PHPT is a disease with a heterogeneous clinical presentation. Patients manifest diverse, often non-specific, symptoms and the results of diagnostic tests are sometimes inconclusive. The present study attempts to identified progression markers of the disease, which would be helpful in assessing the degree of PHPT severity and calcium–phosphate imbalance.

### 4.1. FGF-23

There are few publications concerning the role of FGF-23 in PHPT. To date, most research has focused on CKD (chronic kidney disease) and secondary hyperparathyroidism. In this study, PHPT patients had significantly higher mean concentration of FGF-23 than the persons from the control group. At six months after effective surgery, the protein levels were much lower, but nevertheless higher than in healthy subjects.

Yamashita et al., who investigated concentrations of FGF-23 in the serum of 98 of patients after PTX, obtained different results. The patients were divided into two groups: those with impaired kidney function (creatinine clearance < 70 mL/min) and those with normal kidney function (creatinine clearance ≥ 70 mL/min). The results were compared with assays performed for 104 healthy volunteers. The authors found that the concentrations of FGF-23 were significantly increased only in the group with defective kidney function. Additionally, the assays done in 11 persons on the sixth day post-surgery did not reveal significant changes in the concentration of FGF-23. On the basis of their results, the Japanese authors concluded that the increased values of FGF-23 were merely a consequence of kidney function decline [16]. In the present study, only patients with GFR > 70 mL/min/1.73 m^2^ were taken into account and—although their kidneys were fully functional—significantly higher levels of FGF-23 were detected as compared with healthy subjects.

Nilsson et al. made an attempt to assess FGF-23 plasma concentration as a progression marker of PHPT and its influence on diurnal blood pressure profile. The study comprised 150 patients who had undergone surgical removal of parathyroid adenomas. Blood samples were collected at around six weeks prior to and six weeks after PTX. FGF-23 values significantly declined post-surgery, but there was no control group so the outcomes could not be compared with healthy individuals. The authors emphasised the necessity to continue the research and to measure FGF-23 at further time intervals post-surgery [17].

In the present study, FGF-23 concentrations were determined at six months after treatment. Despite the relatively long time gap and a considerable decrease in FGF-23 values in the study group, these were still significantly higher than the values found in healthy subjects. This may indicate a long-term imbalance in calcium phosphate homeostasis. Because of the need for lengthy observation periods, the utility of FGF-23 as an efficacy marker of surgical treatment is limited. It can be potentially useful as a progression marker for PHPT and the risk of its long-term sequelae. In the current literature on PHPT, no author has attempted to analyse the optimum cut-off point for FGF-23 in this group of patients. From the data gathered in the present study it follows that after excluding other causes of increased FGF-23 levels (e.g., kidney dysfunction), plasma values equal to and higher than 29.8 pg/mL can imply advanced PHPT. However, since the study sample was small our results need to be verified on a larger group of patients.

### 4.2. Klotho

The Klotho protein plays the role of a co-receptor for FGF-23 [18]. Research has been conducted, among other things, on its function in CKD [19], cardiovascular diseases, and in aging processes [20,21]. Regarding PHPT, the few existing studies have focused only on evaluating the expression of Klotho in post-operative samples [22,23,24]. This study is the first to endeavour to assess serum concentrations of Klotho in patients with PHPT. The values we obtained did not differ significantly from those observed in the control group. After effective surgery, the blood concentrations of the protein decreased in a statistically significant way, but because the baseline values were close to those found in healthy individuals, the usefulness of Klotho as a progression marker of the disease is questionable.

### 4.3. Osteocalcin and NTX

The literature on bone-related sequelae of PHPT primarily concentrates on assessing BMD. Less attention is paid to serum concentrations of bone turnover indicators as progression markers of the disease. In this study, we measured serum concentrations of: osteocalcin (bone tissue formation marker) and NTX (bone resorption marker). Statistically significantly higher values of both parameters were found in PHPT patients prior to surgery as compared with the control group. At six months post-surgery, the two indicators were assayed again and a considerable reduction in their concentrations was observed (the values were comparable to those found in healthy individuals).

Tamura et al. examined 11 patients who had parathyroid adenomas removed surgically. The mean serum concentration of osteocalcin at one year after surgery was by 70% lower and remained at a similar level in the following years. The mean urine concentration of NTX was also measured and was found to have declined by 51% from the baseline at a year post-treatment, without significant changes over the follow-up period. Researchers have suggested that bone turnover indicators may potentially be used as evaluation parameters of treatment efficacy in elderly patients [25].

Minisola et al. analysed the concentrations of osteocalcin and urine excretion of NTX in 26 patients after PTX. The study group had significantly higher concentrations of osteocalcin and urine secretion of NTX as compared with healthy subjects. A negative correlation was also found between urine concentrations of NTX and BMD (bone mineral density), in both the lumbar spine and the radius. On that basis, the authors conclude that this parameter is the most useful for assessing bone resorption in PHPT patients [26]. Takami et al. [27] and Carnevale at al. arrived at similar conclusions [28].

As in the case of FGF-23, no researcher has undertaken to identify the optimum cut-off point for osteocalcin and NTX concentrations in this group of patients. The results of our study have demonstrated that in PHPT patients, the optimum cut-off point is 3.65 ng/mL for osteocalcin and 17.07 nM for NTX. Determination of these markers may be beneficial in assessing the intensity of bone remodelling processes, and their normalisation after surgery may be treated as a parameter confirming the efficacy of treatment.

### 4.4. Sclerostin

The canonical (β-catenin dependent) Wnt signal pathway plays an important part in the processes of bone remodelling. Sclerostin is one the antagonists of this pathway: its action causes inhibition of osteoblastogenesis [29,30]. Our research revealed significantly lower concentrations of sclerostin in PHPT patients than in the control group. In patients after PTX, the levels of this glycoprotein were found to have considerably risen in comparison to the levels detected in healthy individuals.

Viapiana et al. [31] examined 21 postmenopausal women with PHPT. The results were compared with those of 42 similarly aged healthy women. None of the patients from the study group had undergone PTX (lack of consent to surgery or contraindications). Similar to our study, a significantly lower mean concentration of sclerostin was detected in PHPT patients than in the healthy controls. The outcomes reported by Viapiana et al. are consistent with those obtained by van Lierop et al. [32], Kaji et al. [33], and Costa et al. [34]. A project by Ardawi et al. comprised 60 PHPT patients who were compared with 74 PTX patients and 268 healthy volunteers. The highest concentrations of sclerostin were detected in the healthy subjects, lower in the post-surgery group, and the lowest in patients with untreated PHPT. Additionally, 27 persons were observed both before and immediately after surgery: from day two to day 360 after PTX. In that group, sclerostin began gradually increasing on the second day post-surgery and returned to normal levels on day 10. What is more, the concentrations of sclerostin were back to the normal range earlier than the other bone turnover markers [35].

The results of the present study are mostly consistent with the outcomes obtained in the above-mentioned paper. It seems likely that the lower level of sclerostin, which inhibits bone tissue formation, observed in PHPT patients is a compensation mechanism. The fact that the levels of this glycoprotein become normalised after effective surgery may make it a potential marker of treatment efficacy.

## 5. Conclusions

The obtained outcomes suggest that osteocalcin and NTX may serve as potential progression markers in PHPT. Additionally, osteocalcin, NTX, and sclerostin might be indicators of PTX efficacy. FGF-23 might prove to be a parameter, which reflects the extent of calcium–phosphate imbalance in PHPT patients, although further research is needed to ascertain its usefulness for monitoring therapeutic effects. Clinical utility of assaying serum concentrations of Klotho in PHPT has not been confirmed.

### Limitations of the Study

The limitation of our study is a small sample group and our findings should be confirmed on a larger cohort.

## Figures and Tables

**Table 1:** Biochemical parameters of study group and control group.

Biochemical Parameters	Study Group				Control Group	
	Before Surgery		After Surgery			
	Mean	SD	Mean	SD	Mean	SD
PTH (pg/mL)	281	139	36	17	40	15
Ca (mmol/L)	2.95	0.19	2.46	0.12	2.41	0.13
P (mmol/L)	0.78	0.18	0.92	0.14	-	-
Creatinine (mg/dL)	0.81	0.16	0.79	0.17	0.75	0.13
eGFR (mL/min/1.73 m^2^)	85.2	12.1	85.5	13.3	88.4	6.7

PTH, parathormone; eGFR, estimated glomerular filtration rate; SD, standard deviation.

**Table 2:** Plasma concentrations of FGF-23 in study group vs. control group.

FGF-23 (pg/mL)	Mean	SD	Min.	Max.	*p*-Value			
Before surgery	57.53	26.31	15.66	106.45	0.016	-		0.004
After surgery	42.78	14.85	9.19	75.22		0.019	b-	
Control group	29.97	13.11	7.82	46.84	-			

**Table 3:** Serum concentrations of Klotho protein in study group vs. control group.

Klotho (ng/mL)	Mean	SD	Min.	Max.	*p*-Value			
Before surgery	1.26	0.84	0.34	3.33	0.001	-		0.288
After surgery	0.62	0.33	0.25	1.46		0.204	b-	
Control group	0.89	0.76	0.32	2.88	-			

**Table 4:** Serum concentrations of osteocalcin in study group vs. control group.

Osteocalcin (ng/mL)	Mean	SD	Min.	Max.	*p*-Value			
Before surgery	7.27	7.78	1.33	42.0	0.043	-		0.046
After surgery	2.11	1.93	0.00	7.36		0.609	b-	
Control group	2.57	1.1	1.35	4.68	-			

**Table 5:** Serum concentrations of NTX (N-terminal telopeptide of type I collagen) in study group vs. control group.

NTX (nM)	Mean	SD	Min.	Max.	*p*-Value			
Before surgery	22.98	9.45	11.0	42.0	0.00007	-		0.003
After surgery	10.36	4.17	5.03	21.15		0.18	b-	
Control group	12.44	2.25	9.1	15.9	-			

**Table 6:** Serum concentrations of sclerostin in study group vs. control group.

Sclerostin (ng/mL)	Mean	SD	Min.	Max.	*p*-Value			
Before surgery	0.44	0.14	0.25	0.77	0.022	-		0.017
After surgery	0.52	0.16	0.29	0.88		0.642	b-	
Control group	0.55	0.18	0.35	0.88	-			

**Table 7:** Spearman’s rank correlation coefficients (significant in bold).

Variable	PTH	Ca	P	FGF-23	Klotho	SCL	OC	NTX
PTH	−	**0.53**	−0.35	0.06	0.41	−0.31	0.18	0.47
Ca	**0.53**	−	−0.17	0.18	0.41	−0.08	**0.49**	**0.62**
P	−0.35	−0.17	−	0.33	−0.14	0.46	0.12	0.11
FGF-23	0.06	0.18	0.33	−	0.39	0.1	0.21	−0.13
Klotho	0.41	0.41	−0.14	0.39	−	−0.19	0.45	0.15
SCL	−0.31	−0.08	0.46	0.1	−0.19	−	−0.42	−0.06
OC	0.18	**0.49**	0.12	0.21	0.45	−0.42	−	0.44
NTX	0.47	**0.62**	0.11	−0.13	0.15	−0.06	0.44	−

SCL, sclerostin; OC, osteocalcin.

## Data Availability

The datasets used and/or analysed during the current study are available from the corresponding author on reasonable request.
